# Supplementary material for: Screen time and chronic neck pain in Peru: A comparative population-based cross-sectional study in the COVID-19 post-pandemic period
Source: PLoS One. 2026 Mar 31;21(3):e0344257. doi: 10.1371/journal.pone.0344257 (PMC13038022; doi:10.1371/journal.pone.0344257)
Supplement: Attachment — Submitted filename: PONE-D-25-60168.docx [file pone.0344257.s002.docx]

The study provides pertinent and timely data on chronic neck pain in Peru two years post-pandemic, yielding significant insights into a public health issue that remains persistent.
Using a sample that is typical of the whole country makes the results more reliable and gives a strong base for making generalizations about the whole population.
Nonetheless, both the exposure and the outcome are self-reported, which creates the potential for recall bias and misclassification; this restriction may have been articulated more explicitly.
The statistical method, especially multinomial logistic regression, is good for the research issue. However, the study would have been better if it had included more confounding factors like job, exercise, and ergonomic problems.
The discussion does a good job of putting the results in the context of worldwide evidence, although some parts are too long and might be shorter to make things clearer.
Despite these limitations, the study provides considerable evidence indicating that extended screen usage is a notable and alterable risk factor for chronic neck discomfort in Peruvian adults.
Overall, the publication meets its main goals and offers useful information to the post-pandemic epidemiological picture. However, future research should aim for more thorough adjustment of confounders and objective metrics.
